# Mixed infections in genotypic drug-resistant *Mycobacterium tuberculosis*

**DOI:** 10.1038/s41598-023-44341-x

**Published:** 2023-10-10

**Authors:** Linfeng Wang, Susana Campino, Jody Phelan, Taane G. Clark

**Affiliations:** 1 Department of Infection Biology, Faculty of Infectious and Tropical Diseases, London School of Hygiene and Tropical Medicine, Keppel St, London, WC1E 7HT UK; 2 Department of Infectious Disease Epidemiology, London School of Hygiene and Tropical Medicine, Keppel St, London, WC1E 7HT UK

**Keywords:** Genomics, Genotype, Bioinformatics, Infectious diseases, Tuberculosis

## Abstract

Tuberculosis disease (TB), caused by *Mycobacterium tuberculosis*, is a major global public health problem, resulting in more than 1 million deaths each year. Drug resistance (DR), including multi-drug (MDR-TB), is making TB control difficult and accounts for 16% of new and 48% of previously treated cases. To further complicate treatment decision-making, many clinical studies have reported patients harbouring multiple distinct strains of *M. tuberculosis* across the main lineages (L1 to L4). The extent to which drug-resistant strains can be deconvoluted within mixed strain infection samples is understudied. Here, we analysed *M. tuberculosis* isolates with whole genome sequencing data (n = 50,723), which covered the main lineages (L1 9.1%, L2 27.6%, L3 11.8%, L4 48.3%), with genotypic resistance to isoniazid (HR-TB; n = 9546 (29.2%)), rifampicin (RR-TB; n = 7974 (24.4%)), and at least MDR-TB (n = 5385 (16.5%)). TB-Profiler software revealed 531 (1.0%) isolates with potential mixed sub-lineage infections, including some with DR mutations (RR-TB 21/531; HR-TB 59/531; at least MDR-TB 173/531). To assist with the deconvolution of such mixtures, we adopted and evaluated a statistical Gaussian Mixture model (GMM) approach. By simulating 240 artificial mixtures of different ratios from empirical data across L1 to L4, a GMM approach was able to accurately estimate the DR profile of each lineage, with a low error rate for the estimated mixing proportions (mean squared error 0.012) and high accuracy for the DR predictions (93.5%). Application of the GMM model to the clinical mixtures (n = 531), found that 33.3% (188/531) of samples consisted of DR and sensitive lineages, 20.2% (114/531) consisted of lineages with only DR mutations, and 40.6% (229/531) consisted of lineages with genotypic pan-susceptibility. Overall, our work demonstrates the utility of combined whole genome sequencing data and GMM statistical analysis approaches for providing insights into mono and mixed *M. tuberculosis* infections, thereby potentially assisting diagnosis, treatment decision-making, drug resistance and transmission mapping for infection control.

## Introduction

Tuberculosis (TB), caused by *Mycobacterium tuberculosis*, is a major global health problem, responsible for 10.6 million cases and 1.6 million associated deaths in 2021 alone^1^. Whilst, TB is a treatable disease, resistance to anti-TB drugs, especially first-line rifampicin (RR-TB) and isoniazid (HR-TB), together called multi-drug resistance (MDR-TB), is making infection control more difficult. To acquire resistance to anti-TB drugs, *M. tuberculosis* drug targets or activating proteins are often mutated^2,3^, including by single nucleotide polymorphisms (SNPs) and insertions and deletions (indels); a process involving vertical, but not horizontal, gene transfer. It is being increasingly recognised that within-host mixed strain infections (MSIs) are contributing to TB drug resistance, with heteroresistance involving the co-existence of susceptible and resistant strains. MSIs can arise due to the reinfection of an infected host with a new strain of *M. tuberculosis*, which is often observed in relapse patients, as well as emerge where there is distinct clonal evolution within the infected host^4^. MSIs may be driven by inadequate treatment schemes where a diagnosed TB patient will receive combination therapies of sometimes toxic drugs for a minimum of 6 months, and non-compliance or treatment failure can arise. Heteroresistance has been responsible for higher rates of treatment failure, thereby limiting treatment options in TB patients^5^. Often without proper strain and drug resistance profiling, the treatment of MSI patients may involve second/third-line drugs with less efficacy, more serious adverse drug reactions, and a prolonged treatment period. Therefore, identifying the complete pathogen diversity within the host is useful for achieving favourable clinical treatment outcomes.

The phylogeny of *M. tuberculosis* consists of 4 major lineages (L1–L4), which consist of different strain types that may vary in their propensity to transmit and cause severe disease^6^. MSIs of *M. tuberculosis* can be identified in high-depth whole genome sequencing (WGS) data through the presence of heterozygous genotypes. Strains and SNPs with high numbers of heterozygous sites are typically removed from the analysis, often thought to be the effects of contamination or sequencing errors. The deconvolution of different lineages within MSIs can be determined from such data by estimating the ratios of allele coverage at different lineage-specific SNPs^6^. However, for heteroresistance the challenges lie in determining the lineage each resistance-linked SNP belongs to; thereby obtaining information for lineage-specific drug resistance profiling in an MSI. To infer this, we often rely on any overlap between the lineage-specific and drug resistance SNPs, which is not straightforward using short-read sequencing data, and often leads to many orphan drug resistance SNPs that are unassigned to strains. However, this problem can be resolved using data from long-sequencing platforms.

**Figure 1:**
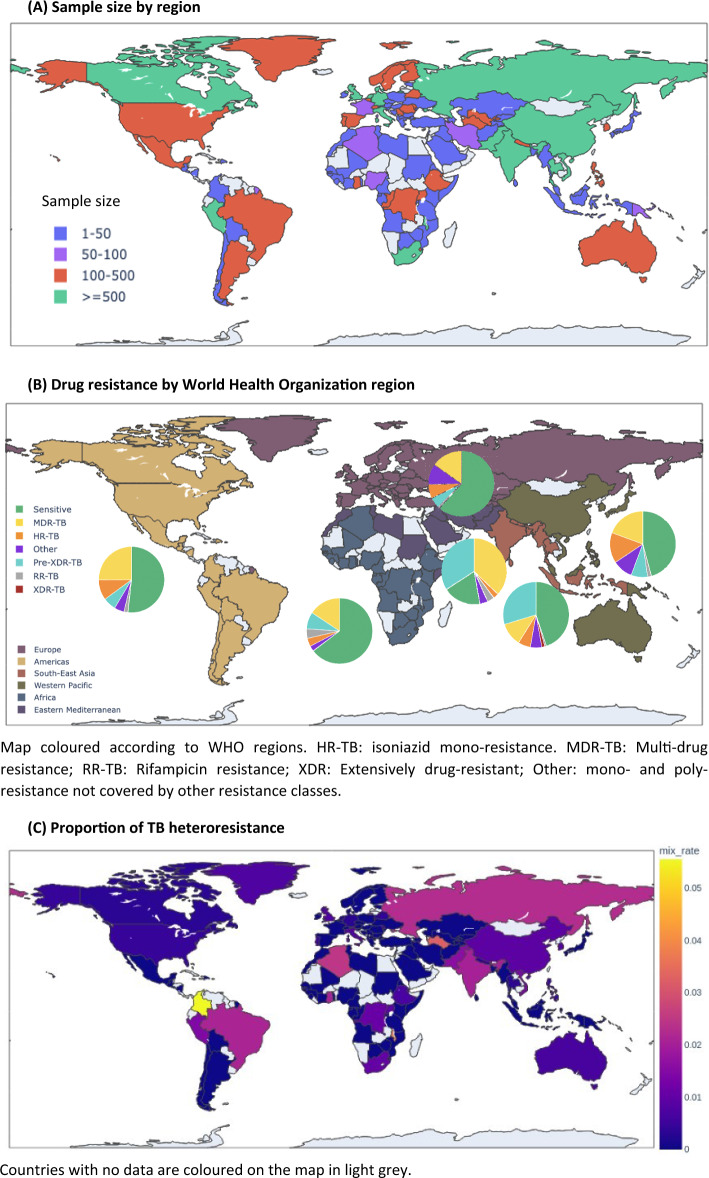
World maps of the 50k *M.* *tuberculosis dataset.* (**A**) Sample size by region. (**B**) Drug resistance by World Health Organization region. Map coloured according to WHO regions. *HR-TB* isoniazid mono-resistance, *MDR-TB* multi-drug resistance, *RR-TB* rifampicin resistance, *XDR* extensively drug-resistant, *Other* mono- and poly-resistance not covered by other resistance classes. (**C**) Proportion of TB heteroresistance. Countries with no data are coloured on the map in light grey.

It is possible to profile drug resistance and lineages from WGS data to inform clinical and infection control, for example, using the TB-Profiler tool^2^. However, whilst it is possible to call mixed genotypes, such software typically lacks the means of disentangling the different SNPs on specific different strains within an MSI, which could enhance profiling. Previous work^4,7,8^ on mixed infections in TB has provided a means of identifying specific lineages involved in MSI samples and the sample drug resistance. Nonetheless, the connection between the identified lineage and sample drug resistance is still undetermined. Here we built a statistical tool based on Gaussian mixture models (GMMs) to distinguish different strain lineages’ fractions in an MSI, and assign drug resistance to each lineage, without the need for detecting drug resistance in lineage-specific SNPs on the same sequencing read. In general, a GMM is a probabilistic model representing multiple Gaussian distributions within a population, and the algorithm determines their number and mixing proportions. Amongst many applications, GMMs have been used successfully to identify protein families^9^, cell types from omics data^10^, and to classify cancers^11^. Here, we apply a GMM model to deconvolute 531 MSI samples detected in a large *M. tuberculosis* WGS "50k" dataset (n = 50,723)^6,12^ by TB-Profiler software^2^ (Fig. 1A). We test the accuracy of the GMM algorithm in a simulation study and estimate the number of MSIs and heteroresistance across different lineages. Ultimately, the disentanglement of strains and drug resistance involved in MSIs could assist in the optimisation of treatment decisions and potentially prevent the emergence of further resistance.

## Results

### Global clinical results

A total of 50,723 *M.* *tuberculosis* isolates with WGS and drug susceptibility test data from 64 countries were analysed, and encompassed all major lineages (L4 48.3%, L2 27.6%, L3 11.8%, L1 9.1%) (Table 1, Fig. 1B). Lineage 2.2.1 (Beijing strain-type) is the most prevalent in four of six World Health Organization (WHO) regions, especially in Southeast Asia (34.5%) and the Western Pacific (51.0%) (Africa 15.6%; Europe 15.9%). Genotypic resistance prediction using TB-Profiler software inferred that 9546 (29.2%), 7974 (24.4%), and 5385 (16.5%) samples were resistant to isoniazid (HR-TB), rifampicin (RR-TB), and at least MDR-TB, respectively (Table 1, Fig. 1B). Across the 18,637 samples with DST data, 3968 (21.3%), 1036 (5.6%) and 8370 (44.9%) were HR-TB, RR-TB, and at least MDR-TB, respectively. The highest level of genotypic drug resistance is in the Eastern Mediterranean region (80.7%), which has predominantly L3 strains (47.4%). L4 strain types, the most globally prevalent strain, were found to be in the largest majority in the Americas (75.6%, globally: 48.3%), with L4.3.3 being the most prevalent sub-lineage (15.6%).

**Table 1:** The *M. tuberculosis* isolates by World Health Organization (WHO) region.

WHO region	N	L1 %	L2 %	L3 %	L4 %	Other* %	Sens %	RR-TB%	HR-TB%	MDR + -TB%	MSI N (%)	Hetero-res %
Africa	8864	4.5	21.2	4.6	64.5	5.2	65.3	4.5	4.0	15.3	1.6	0.5
Americas	6007	5.0	14.0	2.3	75.6	3.1	48.7	1.9	10.0	26.5	1.0	0.7
EM	872	6.7	8.6	54.0	29.1	1.6	19.3	3.4	2.6	36.8	1.1	1.1
Europe	17,017	7.5	16.9	17.1	55.2	3.3	62.8	0.9	6.8	14.5	0.7	0.3
SEA	6176	22.5	40.8	21.6	13.4	1.7	45.0	0.8	6.1	11.4	1.6	1.1
WP	6760	12.9	66.1	0.5	19.6	0.9	45.8	1.8	14.7	19,9	1.1	0.7
Unknown	5027	6.3	27.4	14.4	49.9	2.0	60.4	3.1	9.3	26.7	0	–
Overall	50,723	9.1	27.6	11.8	48.3	3.2	50.0	2.0	7.8	26.9	1.0	1.0

Lineages L1–L4; *Other lineages (L5, L6, L7, La1, *M. bovis*, *M. orygis*); EM Eastern Mediterranean; SEA Southeast Asia; WP Western Pacific; RR-TB rifampicin resistant; HR-TB isoniazid resistant; MDR+-TB refers to MDR-TB, pre-XDR, or XDR resistant; MSI: mixed strain infection; Hetero-res(istance).

### Multiple strain infections

**Figure 2:**
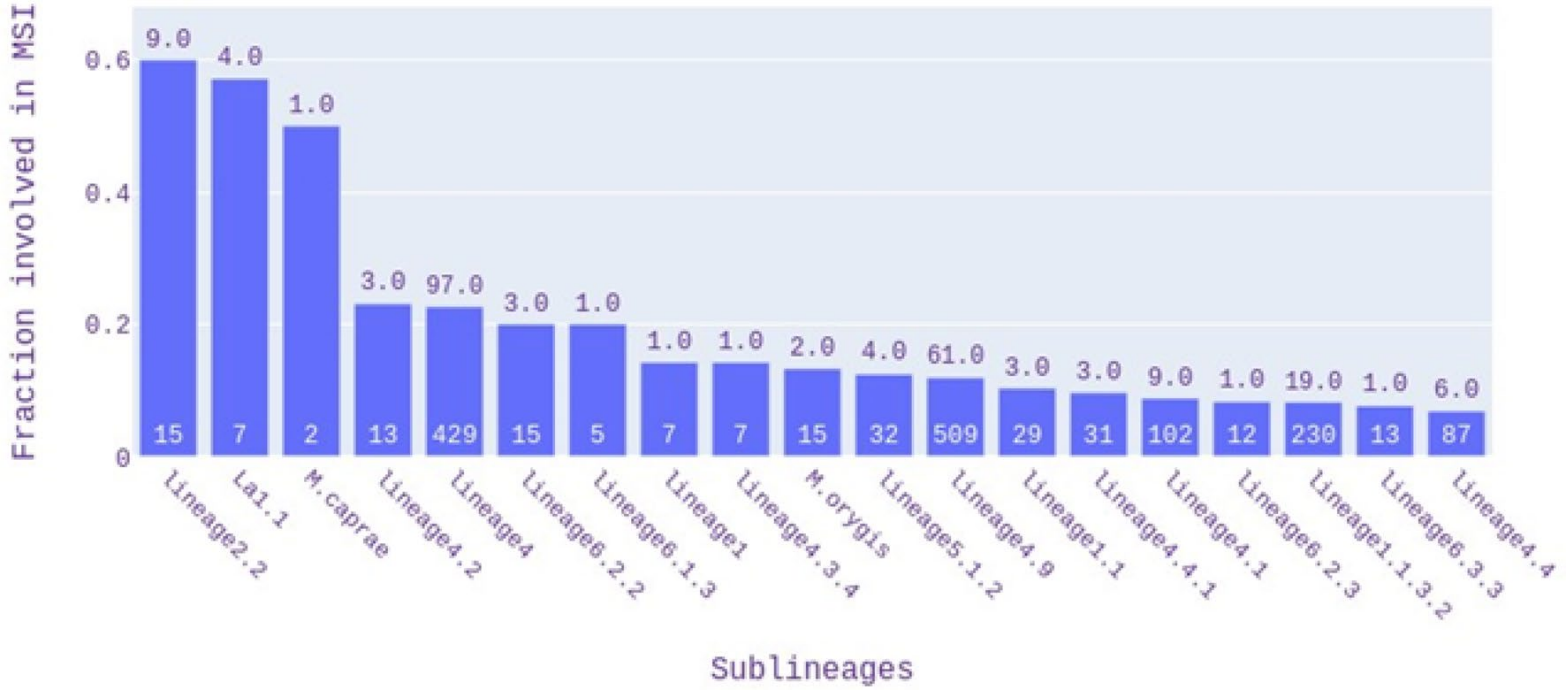
Lineages involved in mixed strain infections (MSIs).

**Figure 3:**
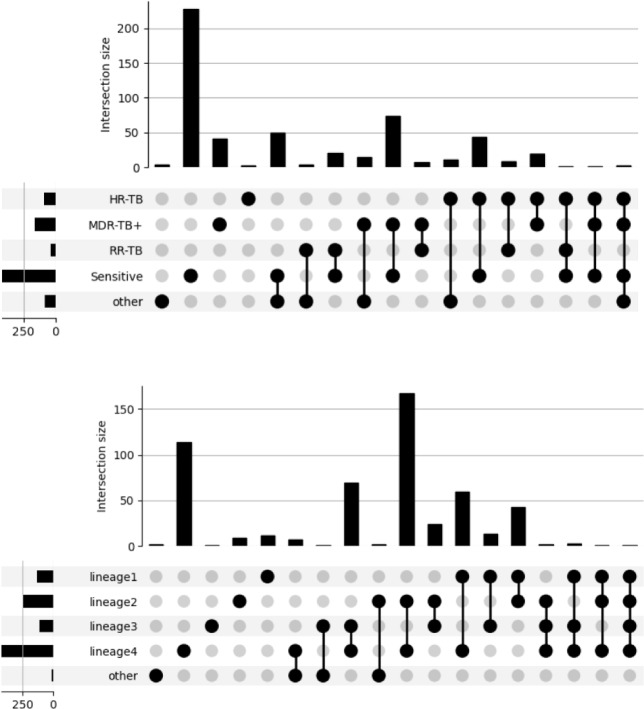
The *M. tuberculosis* isolates putative evidence of mixed strain infections as measured by drug resistance type (n = 531).

Using TB-Profiler software, 531 (1.1%) samples revealed the co-existence of two or more *M. tuberculosis* (sub-)lineages (Table 2, Figs. 2, 3), with some countries having up to 5% of isolates with a MSI (Colombia, Malawi, Turkmenistan, Russia, Brazil, Algeria, and India; Fig. 1C). The vast majority of TB-Profiler determined MSIs could be conferred using Quant-TB software (513/531; 96.6%). Lineage 4 strains (n = 424) were the most frequent strain-types in MSIs, but as a function of sample size, the most prevalent involved L2.2, La1.1, and *M. caprae* (Fig. 2), with the most common combinations involving L4 and L2 (31.3%) strains (Table 2, S1 Table, Fig. 3). The most prevalent drug resistance forms in the MSIs involve isoniazid (170/531; 32.0%), streptomycin (130/531; 24.5%), and ethambutol (73/531; 13.7%) (S2 Table).

**Table 2:** The *M. tuberculosis* isolates with putative evidence of mixed strain infections (n = 531).

Count	L1	L2	L3	L4	Other*
167		X		X	
114				X	
69			X	X	
60	X			X	
43	X	X			
24		X	X		
13	X		X		
12	X				
9		X			
7				X	L5, L6, La1
3	X		X	X	
2					L5, L6
2		X	X	X	
2		X			*M. orygis*, L6
1	X	X		X	
1			X		
1	X	X	X	X	
1			X		*M. bovis*
Overall N (%)	133 (25.0)	249 (46.9)	114 (21.5)	424 (79.8)	14 (2.6)

*Other lineages.

### Performance of GMMs on artificial mixes

The predictive power of a GMM approach was first assessed using WGS data from 48 samples with known mixes of DNA, varying with major proportions of 1, 0.95, 0.9, and 0.7, obtained from clinical Malawi *M. tuberculosis*^4^ strains (S3 Table). GMM models and TB-Profiler achieved low mean squared errors (MSEs) in samples with a predominant strain, but were consistently low overall (GMM: 0.006; TB-Profiler: 0.006). Quant-TB appears to perform better on samples with a major mixing proportion of 0.7, but obtained a higher overall MSE value (0.020) than the other methods.

A limitation of the mixed DNA samples from Malawi is that none are drug resistant and there were no ratios close to parity (50:50). To explore the ability of GMM to predict heteroresistance in a wider range of lineage ratios and larger sample sizes, in silico artificial mixtures with known drug resistance and mixing proportions were generated (S4 Table). Our simulations suggest that the GMM approach achieves high accuracy for the prediction of drug resistance across the minor proportions between 0.05 and 0.50 (accuracy: median 0.93; range: 0.89–0.97). The levels of MSE for the GMM were low and consistent across mixing proportions (overall MSE of 0.012). In comparison, the overall MSE values for TB-Profiler were slightly lower (0.009) and for Quant-TB greater (0.013).

### Application of GMM to clinical isolate data

Having shown that a GMM can estimate mixtures accurately, we applied the approach to the 531 mixed clinical samples in the global dataset. The median number of mixtures was 2 (range: 2–4), and the median major mixing component was 77.6% (range: 50.0–93.7%). Assuming the TB-Profiler mixing prediction to be the gold standard, the GMM had an MSE of 0.007. From the MSIs, 35.4% (188/531) of samples consist of one drug resistant and one sensitive lineage, 21.5% (114/531) consist of only lineages that have drug resistance mutations, and 43.1% (229/531) consist of two lineages that are pan-susceptible.

We present three examples of MSIs that represent the complexity of heteroresistance (Table 3). ERR4796347 is categorised as pre-XDR-TB, which was confirmed using accompanying DST data. From the sequencing coverage, TB-Profiler software found the sample to be a mix of L4.4.3 (64%) and L2.2.1 (Beijing strain; 36%) sub-lineages, which the GMM model confirmed with similar mixing proportions (61%/39%). In addition, the model reveals that lineage 2.2.1 is not responsible for drug resistance mutations, with L4.3.3 being pre-XDR, driven by mutations in genes including *gyrA, rpoB, rrs, fabG1, katG, pncA,* and *embB*. Another sample, ERR4829977, is categorised as MDR-TB, with a mixture of L3 (TB Profiler 88%; GMM 79%) and L2.2.1.1 (TB-Profiler 12%; GMM 21%) strain types. The estimated mixing proportions are reflected in the frequency of drug resistance SNPs, with L3 containing mutations for rifampicin and streptomycin resistance, whilst L2.2.1.1 contains resistance for streptomycin and ethambutol. A further sample, ERR4797884, is categorised as pre-XDR-TB, with the presence of L3.1.2.1 (TB-Profiler 61%; GMM 55%) and L2.2.1 (TB-Profiler 39%; GMM 45%). The GMM analysis revealed that the major L3.1.2.1 strain is isoniazid and streptomycin resistant through *katG* (S315T) and gid (G170*) mutations, respectively, while L2.2.1 also has the same common *katG* mutation and confers all other resistance conferring XDR. This analysis demonstrates the potential for differentiating mixed lineage resistance involving the same mutations.

**Table 3:** Examples of clinical mixed strain infections with disentangled the drug resistance mutations established for each strain using our GMM.

ID	Drug resistance (DR) profile	Strains Major; minor	Major prop.*	Minor prop.*	Major strain DR mutations	Minor strain DR mutations
ERR4796347 (India)	Fluoroquinolones, ethionamide, ethambutol, streptomycin, rifampicin, isoniazid, pyrazinamide	L4.3.3; L2.2.1;	(0.64) [0.61]	(0.36) [0.39]	Ala90Val-gyrA Ser450Leu-rpoB n.514A>C-rrs c.-15C>T-fabG1 Ser315Thr-katG Thr160Ala-pncA Thr100Ile-pncA Met306Ile-embB	
ERR4829977 (Pakistan)	Bedaquiline, clofazimine, ethionamide, ethambutol, isoniazid, rifampicin, streptomycin,	L3; L2.2.1.1	(0.88) [0.79]	(0.12) [0.21]	p.Ile67fs-mmpR5 p.His445Asn-rpoB	p.Lys88Arg-rpsL c.-15C>T-fabG1 p.Gly406Asp-embB
ERR4797884 (India)	Aminoglycosides, capreomycin, ethambutol, ethionamide, fluoroquinolones, isoniazid, kanamycin, linezolid, moxifloxacin, PAS, pyrazinamide, rifampicin, streptomycin	L3.1.2.1; L2.2.1	(0.61) [0.55]	(0.39) [0.45]	Ser315Thr katG Glu170* gid	Asp94Gly gyrA Ser450Leu rpoB Lys43Arg rpsL Cys154Arg rplC n.1484G>T rrs c.-8T>C fabG1 Ser315Thr katG Thr177Pro pncA c.-16C>T thyX Met306Val embB

*Mixing proportions: (TB-Profiler prediction) [GMM prediction]; PAS P-aminosalicyclic acid.

## Discussion

Mixed-strain infections (MSIs) of *M. tuberculosis* present themselves in WGS data as heterozygous genotypes and are typically removed from the analysis. However, they are informative for heteroresistance, which can de-rail treatment effectiveness. The degree of MSIs may have been historically underestimated with various colony sampling techniques leading to the culture of clonal *M. tuberculosis*, as well as through bioinformatic analyses that have excluded samples with too many heterozygous genotypes. Recently, it has been found that short-term culture methods and the direct WGS of sputum or lung tissue can lead to a more accurate representation of within-host *M. tuberculosis* diversity of TB patients^13^. Relatedly, studies of lung tissues reveal that TB infections may be more complex than previously thought, compared to sputum, which is typically the predominant biological sample used. Further, it has been shown that the magnitude of MSIs in high-burden TB settings is underestimated when only testing sputum samples^14^, and in such settings diversity and complexity can be further reduced and underestimated through *M. tuberculosis* culture and colony selection^15^.

In other contexts, such as malaria, the degree of the multiplicity of infection may be a surrogate of transmission intensity^16^. A previous TB study has identified MSIs in high transmission regions in Pakistan^12^, and we confirm an example of such a complex sample (ERR4829977). In our work, we observe the higher involvement of lineages 2 and 4 in MSIs, which may reflect the convenience nature and confounding effects of the sampling, but such strain types have been found previously to be more transmissible and virulent^6^. For samples in lineages 5 and 6, which are thought to be less transmissible and have slower growth on conventional TB diagnostic media that may influence phenotypic testing results, our genotypic-based modelling approach could detect MSIs. Overall, our results appear to reveal decreased involvement of the less transmissive lineages, such as lineage 7, which is consistent with other studies^17^, but may also be due to their lower sequencing rates. Irrespective, none of the MSI samples found seemed to be present in transmission cluster, which may be indicative of re-infection of TB by incomplete treatment and poor adherence.

Typically, the issue of MSIs can be disentangled into linked components, namely, identifying their presence, followed by estimation of the (minimum) number of clones, and then deconvoluting the genotypes in those clones. Here, we applied a GMM method to determine the clonal drug-resistant genotypes in MSIs in *M. tuberculosis* with WGS data, identified initially by TB-Profiler^4^, which has the flexibility of using different informative mutation lists for genotypic profiling. It was found that the signal from alternative haplotype frequency was sufficient to differentiate strains, supported by the analysis of mixtures in artificially generated mixed samples. Similarly, Quant-TB and TB-Profiler software were used to confirm the mixing proportions, and the GMM offered lower or comparable error rates in simulated data and samples. Overall, the GMM approach appears to provide a “rapid”, non-culture-based method to assign the drug resistance profile to each lineage, thereby providing insights into lineage-specific drug resistance, which can inform clinical decision making. Specifically, the disentanglement of drug resistance to each lineage in an MSI can assist diagnosis and optimise the personalisation of treatments. It can also help prevent the development of drug resistance, including by avoiding the use of ineffective drugs. Our study, therefore, provides additional proof of concept evidence for the use of WGS-based diagnostics.

Our GMM approach can be used to monitor the within person evolution of strains, the detection of drug resistance mutations and their transmission or related outbreaks at a population level, where tracking the source and spread of each strain can be challenging. It can detect and dissect susceptible samples from the same patient with similar or identical sub-lineages, thereby potentially inferring reinfections. If these samples were from an MSI, and not with identical genomes, our approach would work directly. The detection of heteroresistant infections within the same sub-lineage is more difficult, but can also be analysed, as our input files contain the proportion of alternative allele coverage in SNPs, which can be used for detecting any mixed gene sequencing reads.

Our approach was tested using a convenience sampled collection of isolate data, sourced mostly from clinical samples collected across many different studies using varying individual collection and laboratory culture methods (e.g., MGIT), which may have influenced the estimated prevalence of MSIs and drug resistance. The growing application of whole genome or amplicon-based sequencing platforms, including using portable Oxford Nanopore Technology, will lead to increasing amounts of genomic data for such surveillance and clinical applications, including an accurate estimation of the extent of MSIs Further improvements to the GMM approach can be made by using the *M. tuberculosis* phylogenetic tree structure to extend the approach to other members of the MTBC, as well as exploit intrinsic linkage disequilibrium patterns to increase the strain lineage identification for each drug resistance SNP. In addition, it has been shown that some strains of *M. tuberculosis* are preferentially associated with resistance to certain drugs^18^, and mixed resistance accuracy could be improved by including this association. Alternatives or extensions to GMMs, such as variational GMM, can also be implemented to improve the levels of performance. In lieu of such efforts, we have presented a GMM-based tool that uses WGS data to disentangle lineage and drug resistance genotypes, which can be used to inform clinical and surveillance decision-making for TB control.

## Methods

### Clinical isolates and sequence analysis

*M. tuberculosis* isolates (n = 50,723) with publicly available WGS and drug susceptibility test (DST) data were analysed^6,12^. The sequencing read data were generated using Illumina next-generation sequencing (NGS) technology. Samples with > = 99% genome-wide coverage and sequencing read depths of 30-fold or higher were accepted. These isolates covered all the main lineages (Table 1). Raw read sequences were trimmed by trimmomatic software^19^. BWA-MEM software (v0.7.17-r1188) was used to process and align trimmed reads to the H37Rv reference sequence (Genbank: NC 000962.3). BCFtools (v1.14) and GATK software^20^ (v4.1.3.0) using the HaplotypeCaller function (parameters: -ERC GVCF) were then used to identify SNPs^21^. Monomorphic SNPs and those in highly variable *pe/ppe* genes were removed. TB-Profiler software^2^ was used to infer genotypic drug resistance and detect MSIs, specifically, the sub-lineages and their supported read coverage within each sample. Quant-TB software^7^ (default parameters) was run to confirm the MSIs found by TB-Profiler.

### Gaussian mixture model

A GMM was built for each sample using Scikit-learn^22^ and applied to the ratios of alternative to total allele counts across SNPs contained in a variant calling file (vcf format). This flexible format can be used for detecting any mixed gene reads and identify MSIs between and potentially within sub-lineages. Our approach is summarised (S1 Figure). The GMMs could contain any number of mixture components, as the ‘multi’ option was applied. The outputs for each sample include the number of mixture components, their parameters (mean, standard deviation (SD)), confidence in the mixtures (−/+ 1 SD from the mean), and their mixing proportions. Each SNP is placed within the Gaussian distributions, allowing inference on the assigned strain (component) and its associated confidence (e.g., a probability), leading to a delineation of individual strains and their drug resistance patterns.

### Assessing the performance of the GMM

The performance of the GMM approach was assessed by simulating artificial mixtures from empirical data. Artificial mixtures were created using Seqtk software (v1.3-r106) (https://github.com/lh3/seqtk) across 240 mixture simulations obtained from a combination of 8 clinical samples with majority/minority strain proportions ranging from 0.50/0.50 to 0.95/0.05 in 0.05 increments, covering within and between mixes of L1 to L4. The variant calling for generating sample-specific vcf file generation was performed using Freebayes software (v1.3.5)^23^ Measures of performance included the mean square error (MSE) calculated from the predicted and actual lineage ratios (using the Scikit-learn package), and the accuracy of the drug resistance profiling compared to TB-Profiler predictions. The GMM performance was also assessed using WGS data from a set of 48 mixed *M. tuberculosis* DNA samples that were generated in vitro from clinical cultures of Malawi patients with known mixing proportions^4^. Further, as a comparison, we used Quant-TB software (default settings) to estimate the mixing proportions.

### Supplementary Information

Supplementary Information 1.

Supplementary Information 2.

## Data Availability

The clinical datasets analysed during the current study are available in the EMBL-EBI repository, The accession number for the samples can be found in the supplementary file supplementary_data1. The code for the command line tool Gaussian mixed model for TB (GMM4TB) is available on GitHub (https://github.com/linfeng-wang/GMM4TB).
