# Supplementary material for: Mixed infections in genotypic drug-resistant Mycobacterium tuberculosis
Source: Sci Rep. 2023 Oct 10;13:17100. doi: 10.1038/s41598-023-44341-x (PMC10564873; doi:10.1038/s41598-023-44341-x)
Supplement: Supplementary file 2 — Supplementary Information 2. [file 41598_2023_44341_MOESM2_ESM.pdf]

SUPPLEMENTARY INFORMATION

S1 Figure

The approach: GMM Gaussian Mixture model

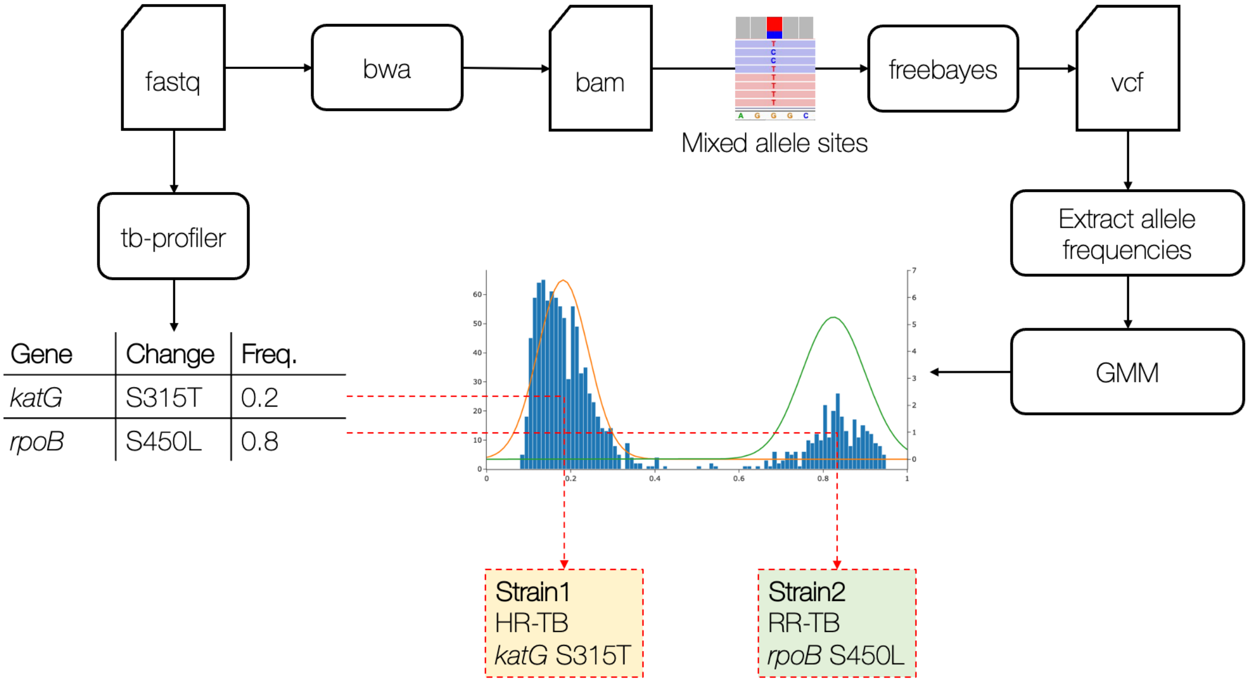

S1 Table

Common combinations of sub-lineages in the MSIs (n=207/531)

(Sub-)lineage	(Sub-)Lineage	Count
L4	L2.2.1	28
L4.4.1.1	L2.2.1	17
L4.3.3	L2.2.1	16
L2.2.1	L1.1.1.1	16
L3	L2.2.1	14
L4	L3	12
L4.5	L2.2.1	9
L2.2.1	L1.1.2	9
L4.3.3	L4.1.2.1	8
L4.1.2.1	L2.2.1	7
L4.8	L2.2.1	7
L4	L1.2.2.2	6
L4.3.3	L3	6
L2.2.2	L2.2.1	6
L1.2.2.2	L1.1.2	6
L4.5	L4.4.2	5
L4	L3.1.1	5
L4.1.2.1	L4.1.1	5
L2.2.1	L1.1.1	5
L3	L1.1.2	5
L4.4.2	L2.2.1	5
L4.8	L4.3.3	5
L4.2.1	L2.2.1	5

S2 Table

The drug resistance profiles of the *M. tuberculosis* isolate with putative evidence of mixed strain infections (n=531)

Count	Sensitive	MDR+	HR-TB	RR-TB	Other*
220	X				
74	X	X			
50	X				ethambutol, ethionamide, kanamycin, cycloserine, ofloxacin, para-aminosalicylic acid, pyrazinamide, streptomycin
44	X		X		
41		X			
20	X			X	
19		X	X		
15		X			ethambutol, ethionamide, kanamycin, ofloxacin, para-aminosalicylic_acid, pyrazinamide, streptomycin
11			X		ethionamide, kanamycin, ofloxacin, para-aminosalicylic acid, streptomycin
8			X	X	
7		X		X	
7	X				
4				X	ethambutol, ofloxacin, pyrazinamide, streptomycin
4					capreomycin, ethambutol, ofloxacin, ethionamide, streptomycin
3			X	X	
2	X	X	X		
1	X	X	X		ethambutol, kanamycin, ofloxacin, streptomycin, ethambutol
1	X		X	X	
Overall N (%)	451 (85.0%)	145 (27.3%)	90 (16.9%)	31 (5.9%)	76 (14.3%)

HR-TB: isoniazid mono-resistance. RR-TB: Rifampicin resistance; MDR-TB+: Multidrug resistance and above, including extensively drug-resistant; Other: drug resistances specified in the column found in the contributing lineage of the mixture.

S3 Table

Performance of the GMM and alternative approaches on 48 mixtures of Malawi *M. tuberculosis* DNA

Strain mix ratio*	N	GMM MSE	TB-Profiler MSE	Quant-TB MSE
30:70	12	0.0175	0.0158	0.0078
10:90	12	0.0046	0.0044	0.0074
5:95	12	0.0031	0.0024	0.0282
0:100	12	0.0000	0.0000	0.0367
Overall	48	0.0063	0.0057	0.0200

* Minor: Major; MSE mean squared error

S4 Table

Performance of the GMM and alternative methods on 240 artificial *in-silico* mixtures with drug resistance (DR) mutations

Strain mix ratio*	N	GMM MSE	GMM DR Accuracy	TB-Profiler MSE	Quant-TB MSE
5:95	24	0.0208	0.9229	0.0044	0.0015
10:90	24	0.0155	0.9554	0.0062	0.0047
15:85	24	0.0146	0.8919	0.0086	0.0067
20:80	24	0.0168	0.8979	0.0104	0.0091
25:75	24	0.0157	0.9239	0.0105	0.0150
30:70	24	0.0128	0.9279	0.0090	0.0149
35:65	24	0.0088	0.9270	0.0065	0.0140
40:60	24	0.0044	0.9662	0.0102	0.0129
45:55	24	0.0035	0.8878	0.0182	0.0171
50:50	24	0.0076	0.9286	0.0044	0.0264
Overall	240	0.0121	0.9230	0.0088	0.0122

* Minor: Major; MSE mean squared error
